# The Number of Stenotic Intracranial Arteries Is Independently Associated with Ischemic Stroke Severity

**DOI:** 10.1371/journal.pone.0163356

**Published:** 2016-09-20

**Authors:** Xiaodan Wei, Zhuang Liu, Min Li, Chunhua Yang, Wenming Wang, Xianglin Li, Shuping Zhang, Xuri Li, Geng Tian, Jonas Bergquist, Bin Wang, Jia Mi

**Affiliations:** 1 Medicine and Pharmaceutics Research Center, Binzhou Medical University, Yantai, Shandong, China; 2 Department of Clinical Imaging, Affiliated Hospital, Binzhou Medical University, Binzhou, Shandong, China; 3 Department of Chemistry-BMC and SciLifeLab, Analytical Chemistry, Uppsala University, Uppsala, Sweden; Jilin University, CHINA

## Abstract

**Background:**

The severity of ischemic stroke symptoms varies among patients and is a critical determinant of patient outcome. To date, the association between the number of stenotic intracranial arteries and stroke severity remains unclear.

**Aims:**

We aimed to investigate the association between the number of stenotic major intracranial arteries (NSMIA) and ischemic stroke severity, as well as the degree of stenosis and common stroke risk factors.

**Methods:**

We performed a retrospective analysis of patients with digital subtraction angiography (DSA)-confirmed ischemic stroke. Clinical stroke severity was measured using the National Institutes of Health Stroke Scale (NIHSS). The number of stenotic vessels was counted from the internal carotid arteries and vertebral arteries, bilaterally.

**Results:**

Eighty three patients were recruited from a single center and included in the study. NSMIA was significantly correlated with stroke severity (*Pearson Correlation Coefficient* = 0.485, *P* < 0.001), but not with the degree of stenosis (*Pearson Correlation Coefficient* = 0.01, *P* = 0.90). Multivariate regression analysis revealed that NSMIA was significantly associated with the NIHSS score after adjusting for stroke risk factors. The adjusted odds ratio (per lateral) was 2.092 (95% CI, 0.865 to 3.308, *P* = 0.001). The degree of stenosis was also significantly associated with the NIHSS score after adjusting for common risk factors. The odds ratio (per 10%) was 0.712 (95% CI, 0.202 to 1.223, *P* = 0.007).

**Conclusions:**

The number of stenotic intracranial major arteries is associated with the severity of ischemic stroke independent of the degree of stenosis and other stroke risk factors. To the best of our knowledge, this has not been previosuly studied in great detail using DSA. Our data highlight the importance of examining all major arteries in stroke patients.

## Introduction

Ischemic stroke represents an estimated 80% of all stroke cases. The clinical manifestations of ischemic cerebrovascular disorders vary from asymptomatic to fatal strokes. The severity of the initial stroke symptoms strongly predicts the response to therapy and subsequent patient outcome [1]. Therefore, it is crucial to investigate the factors associated with ischemic stroke severity. Currently, several factors have been reported to be associated with symptom severity, among which the degree of stenosis of the cerebral arteries is considered the most predictive [2]. Other factors reported to be associated with stroke severity include the location of the stenosis [3], plaque morphology [4], arterial calcification [5] and the presence of collateral circulation [6, 7]. However, the association between the number of stenotic arteries and the stroke symptom severity remains unclear. Bilaterally, the internal carotid arteries (ICA) and vertebral arteries (VA) are the four major vessels which supply blood flow to the brain. Ischemic stroke patients can present with stenosis of more than one single artery. Bilateral ICA stenosis and bilateral VA stenosis have been frequently reported [8, 9]. However, the concept of multiple vessel disease had been ignored for a long time until recent findings from a population based study identified “three-vessel-disease” in 24% of patients with VA stenosis [10]. For patients with bilateral or multilateral stenosis, the importance of the number of stenotic arteries has not been fully assessed. The association between the number of stenotic vessels and the severity of clinical symptoms, as well as other stroke risk factors remains unclear.

We hypothesized that the number of stenotic arteries is a novel factor associated with the severity of ischemic cerebrovascular disease. In this study, we defined this novel factor as the number of stenotic major intracranial arteries (NSMIA), and investigated the association between this and stroke severity, the degree of stenosis, and other common stroke risk factors. Ischemic stroke severity was assessed using the National Institutes of Health Stroke Scale (NIHSS) [11], and the stenosis status of the four major cerebrovascular arteries of each patient was examined by digital subtraction angiography (DSA).

## Methods

### Patients Enrollment

We performed a retrospective analysis of 83 patients with ischemic stroke symptoms who presented to the Department of Clinical Physiology, Binzhou Medical University affiliated Hospital, Shandong, China, between December 1, 2012 and December 1, 2013. Patients with atrial fibrillation, cerebral hemorrhage, subarachnoid hemorrhage, serious liver and kidney disease were excluded. Stroke severity was assessed using the NIHSS score on admission by trained clinical nurses who were blinded to the study design. Plasma samples were obtained after an overnight fasting and analyzed in the hospital’s central laboratory using a chemical analyzer according to standard clinical analysis protocols (AU2700 Beckman Coulter, Fullerton, CA). The triglyceride, total cholesterol, low density lipoprotein, fasting blood glucose and other indicators were measured. All of the subjects underwent a computed tomography angiography (CTA) to confirm the ischemic stroke (data not shown), and further referred to DSA for consideration of revascularization therapy. All subjects provided written informed consent. The results of this study did not affect clinical decision-making. The research protocol was proved by the Binzhou Medical University Medical Ethics Committee.

### Imaging

The DSA procedure was performed on a Siemens AXIOM Artis system (Erlangen, Germany) according to standard protocol. DSA images were obtained by two experienced sonographers working independently who were blinded to the patients’ clinical symptoms and the experimental design. Post-processing images were used to evaluate the degree of intracranial artery stenosis. Four major large intracranial vessels including the ICA and VA bilaterally were evaluated. Conventional artery stenosis was calculated according to angiographic criteria with the method used in NASCET [12]. Arterial stenosis is defined as narrowing of more than 20%. For patients with more than one stenotic artery, the degree of stenosis was recorded from the most severe artery. The novel factor NSMIA was defined as the total number of arteries with stenosis from two pairs of ICA and VA.

### Statistical Analysis

Statistical analysis was performed using statistics analysis software SPSS v20 (IBM SPSS, Chicago, IL). Patient demographics and clinical characteristics were summarized as mean ± standard deviation for continuous data and as number (percentage) for the categorical data. A univariate analysis was performed to evaluate the differences in NIHSS score and the degree of stenosis between different NSMIA categories. The difference between the NIHSS score and the degree of stenosis between groups was evaluated with one-way ANOVA test. To investigate the association between NSMIA and stroke severity and other risk factors, a bivariate correlation analysis was performed on NSMIA against NIHSS, the degree of stenosis and common stroke risk factors including age, sex, the degree of stenosis, body mass index (BMI), type 2 diabetes mellitus (T2DM), previous cardiovascular disease (CVD), smoking status, systolic blood pressure (SBP), blood glucose, total cholesterol, high density lipoprotein cholesterol (HDL-C), low density lipoprotein cholesterol (LDL-C) and triglyceride levels. Data was shown as the median (minimum–maximum). The association between NSMIA and NIHSS score and continuous stroke risk factors was assessed with the Pearson correlation coefficient. Point biserial correlation coefficient was used to assess the association between NSMIA and categorical stroke risk factors. A multivariate linear regression model was constructed to examine the association between NIHSS score and multiple covariates. The NIHSS score was set as the primary dependent variable. Joined independent variables included age, sex, degree of stenosis, NSMIA, BMI, T2DM, smoking status, SBP, blood glucose, total cholesterol, HDL-C, LDL-C, and triglyceride levels. A Two-tailed *P*<0.05 was considered statistically significant.

## Results

### Demographic and Clinical Characteristics

A total of 83 patients were enrolled in the study. Participant characteristics are shown in Table 1. The mean (SD) age of the subjects was 59.6 (12.2) [range, 23–78]. 60.2% (n = 50/83) of the cohort was male. The median NIHSS score of the population was 10 [range. 1–15]. Among all patients, 47 (56.6%) presented with unilateral stenosis, 21 (25.3%) presented with bilateral stenosis and 15 (18.1%) presented with multilateral stenosis. The multilateral stenosis group included 11 patients (13.2%) who sustained three-vessel stenosis and 4 patients (4.8%) with four-vessel stenosis. In the total cohort, the mean (SD) degree of stenosis was 84.1% (19.4%) [range, 20%–99%].

**Table 1:** Baseline Demographic and Clinical Characteristics for Total Patient Population.

Characteristics	Total (n = 83)
NIHSS,median[range]	10 [1–15]
Age, mean(SD),[range],y	59.6(12.2)[23–78]
Sex,Male (%)	50 (60.2)
BMI, mean(SD), kg/m^2^	25.02(2.38)
DM (%)	16 (19.2)
Smoking status (%)	21 (25.3)
Family history (%)	14 (16.9)
SBP, mean(SD),mm Hg	153.4 (26.6)
DBP, mean(SD),mm Hg	88.9 (12.6)
Stenosis profiling	
Degree of Stenosis, mean(SD) [range]	84.1% (19.4%) [20%-99%]
Number of arteries with stenosis	
Unilateral stenosis (%)	47 (56.6)
Bilateral stenosis (%)	21 (25.3)
Three vessels stenosis (%)	11 (13.3)
Four vessels stenosis (%)	4 (4.8)
Biochemical	
Glucose, mean(SD), mmol/L	6.02(1.95)
Total Cholesterol, mean(SD), mmol/L	4.83(1.04)
Triglyceride, mean(SD), mmol/L	1.52(0.63)
HDL-C, mean(SD), mmol/L	1.08(0.21)
LDL-C, mean(SD), mmol/L	2.87(0.91)

Abbreviations: NIHSS, National Institutes of Health Stroke Scale; SD, Standard Deviation; BMI, Body Mass Index; DM, Diabetes Mellitus; SBP, Systolic Blood Pressure; DBP, Diastolic Blood Pressure; HDL-C, High Density Lipoprotein Cholesterol; LDL-C, Low Density Lipoprotein Cholesterol

### Univariate Analysis

**Fig 1:**
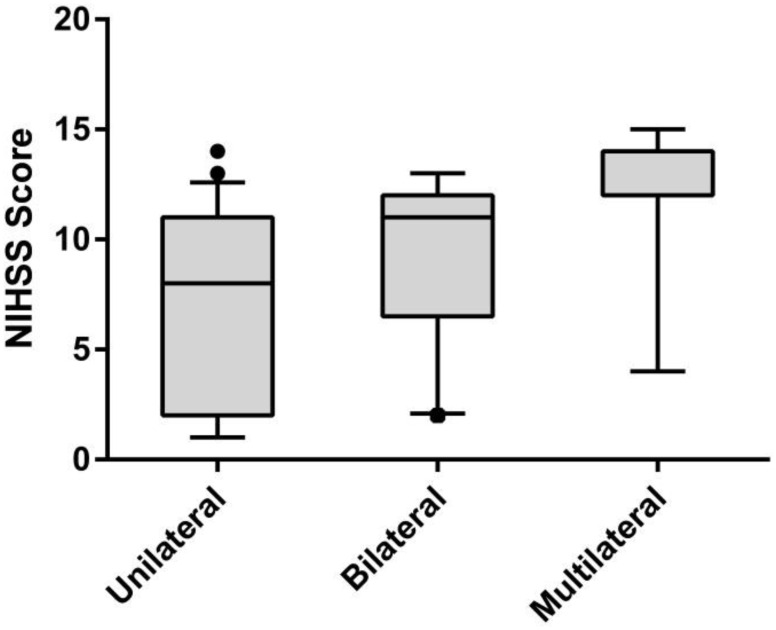
Variation of National Institutes of Health Stroke Scale (NIHSS) scores with different stenotic lateral number categories. unilateral (n = 47), bilateral (n = 21) and multilateral (n = 15); median NIHSS(unilateral) = 7, median NIHSS(bilateral) = 11 and median NIHSS(multilateral) = 13, *P*<0.001.

**Fig 2:**
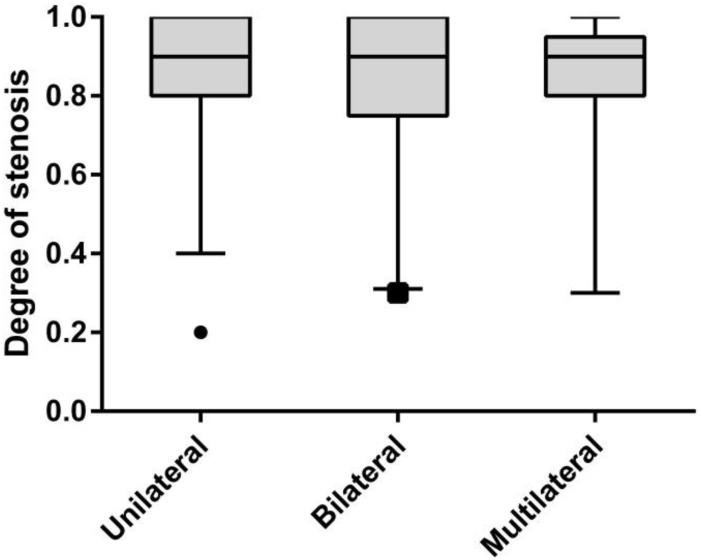
Variation of degree of stenosis with different stenotic lateral number categories. unilateral (n = 47), bilateral (n = 21) and multilateral (n = 15); median degree of stenosis (unilateral) = 90%, median degree of stenosis (bilateral) = 90% and median degree of stenosis (multilateral)90%, *P* = 0.95.

Due to the limited number of patients with four-vessel disease (n = 4), the NISMA was dichotomized into three categories: unilateral (NISMA = 1, n = 47), bilateral (NISMA = 2, n = 21) and multilateral (NISMA = 3 & 4, n = 15). The NIHSS score was significantly different between these categories (median NIHSS, 7 vs. 11 vs. 13, *P*_*trend*_ < 0.001), as shown in Fig 1. No significant difference in the degree of stenosis was observed among the three categories (median degree of stenosis, 90% vs. 90% vs. 90%, *P*_*trend*_ = 0.95), as shown in Fig 2.

### Bivariate Correlation analysis

The correlation results are presented in Table 2. NSMIA was strongly correlated with the NIHSS score (*r* = 0.485, *P* < 0.001, 95% CI 0.342–0.609), age (*r* = 0.415, *P* < 0.001, 95% CI, 0.273–0.546), and SBP (*r* = 0.501, *P* < 0.001, 95% CI, 0.248–0.683). The degree of stenosis was not correlated with the NSMIA (*r* = 0.01, *P* = 0.90, 95% CI, -0.168 to 0.178)

**Table 2:** Bivariate Correlation Analysis of NSMIA with NIHSS and Common Stroke Factors.

Variable	Correlation coefficient	*P* value	95% CI
Age	0.415	<0.001	0.273 to 0.546
Sex	0.06	0.059	-0.16 to 0.278
NIHSS	0.485	<0.001	0.342 to 0.609
Degree of stenosis	0.014	0.90	-0.168 to 0.178
BMI	0.193	0.08	0.012 to 0.35
DM	0.222	0.04	0.001 to 0.463
Previous CVD	0.107	0.34	-0.038 to 0.272
Smoking status	0.003	0.98	-0.169 to 0.205
Family history	0.282	0.01	0.029 to 0.509
SBP	0.501	<0.001	0.248 to 0.683
Glucose	0.09	0.42	-0.125 to 0.364
Total Cholesterol	0.056	0.61	-0.185 to 0.136
Triglyceride	0.061	0.58	-0.049 to 0.161
HDL-C	0.058	0.60	-0.179 to 0.135
LDL-C	-0.045	0.69	-0.258 to 0.113

Abbreviations: NIHSS, National Institutes of Health Stroke Scale; NSMIA, Number of Stenotic Major Intracranial Arteries; BMI, Body Mass Index; DM, Diabetes Mellitus; CVD, Cardiovascular Disease; SBP, Systolic Blood Pressure; HDL-C, High Density Lipoprotein Cholesterol; LDL-C, Low Density Lipoprotein Cholesterol

### Multivariate Regression Analysis

The result of the multivariate regression analysis is provided in Table 3. Four factors are significantly and independently associated with the NIHSS score. The most significant independent factors were NSMIA and the degree of stenosis. The Odds Ratio (OR) for NSMIA (per stenotic lateral) to NIHSS after adjusting for all other factors was 2.092 (95% CI, 0.865–3.308, *P* = 0.001). The OR for the degree of stenosis (per 10%) to NIHSS was 0.712 (95% CI, 0.202–1.223, *P* = 0.007). Two other modest significant predictors were female gender (OR = 2.868, 95% CI, 0.622–5.114, *P* = 0.01) and HDL-C (OR = -6.571 (per 1 mmol/L), 95% CI, -11.959 to -1.183, *P* = 0.02).

**Table 3:** Multivariate linear regression of NIHSS on stenosis profiling and common stroke risk factors.

Variables	Estimate (SE)	*P* value	95% CI
Age (per y)	0.016(0.046)	0.74	-0.076 to 0.107
Sex	2.868(1.125)	0.01	0.622 to 5.114
NSMIA (per 1 lateral)	2.098(0.618)	0.001	0.865 to 3.331
Degree of Stenosis (per 10%)	0.712(0.2556)	0.007	2.02 to 1.223
BMI (per 1 kg/m^2^)	-0.116(0.214)	0.59	-0.543 to 0.312
DM	-2.303(1.725)	0.19	-5.747 to 1.141
Previous CVD	-3.658(1.951)	0.07	-7.552 to 0.235
Smoking	-0.114(1.163)	0.92	-2.435 to 2.207
Family history	-0.374(1.295)	0.77	-2.957 to 2.210
SBP (per 1mm Hg)	0.021(0.021)	0.31	-0.020 to 0.062
Glucose (per 1mmol/L)	0.007(0.339)	0.98	-0.669 to 0.682
Total Choesterol (per 1mmol/L)	0.309(0.911)	0.74	-1.509 to 2.126
Triglyceride (per 1mmol/L)	0.318(0.884)	0.72	-1.446 to 2.083
HDL-C (per 1mmol/L)	-6.571(2.700)	0.02	-11.959 to -1.183
LDL-C (per 1mmol/L)	-1.119(0.905)	0.22	-2.926 to 0.687

Abbreviations: NIHSS, National Institutes of Health Stroke Scale; NSMIA, Number of Stenotic Major Intracranial Arteries; CI, Confidence interval; BMI, Body Mass Index; DM, Diabetes Mellitus; Cardiovascular Disease; SBP, Systolic Blood Pressure; HDL-C, High Density Lipoprotein Cholesterol; LDL-C, Low Density Lipoprotein Cholesterol

## Discussion

In the present study, we investigated the association between the number of stenotic major intracranial arteries and the initial ischemic stroke severity. To the best of our knowledge, there is no previous report regarding the importance of the number of stenotic arteries in patients with cerebrovascular disease. Our results illustrate that the NSMIA is strongly associated with ischemic stroke severity. This association remains statistically significant after adjusting for the degree of stenosis and other common risk factors. Thus, we suggest that the NSMIA is a novel independent factor associated with the severity of ischemic stroke. The degree of stenosis and the NSMIA are thus two uncorrelated and independent factors related to the severity of ischemic stroke. In patients with multilateral stenosis, two independent factors which should be assessed when determining stroke severity are the number of stenotic arteries and the degree of stenosis from the most severe artery. Taking both of these into consideration can partially explain the discrepancy between stenosis severity and symptoms severity, especially in those patients presenting with mild degrees of stenosis in multiple vessels.

In our study, we also prove that multi-vessel disease is prevalent in the ischemic stroke cohort. The reports on multi-vessel disease are very limited due to the unawareness of this correlation. The only current report is from the Oxford Vascular study published by Marquardt and colleagues [10], in which 9 of the 37 patients identified with VA stenosis had three-vessel disease [10]. Based on our population, we report a prevalence of multi-vessel disease in the ischemic stroke cohort of 18% (n = 15/83). The difference between our value and that stated in the previous study may be due to the fact that the latter focused on patients in the VA stenosis cohort while we report from a general ischemic stroke cohort. Both of the studies confirmed the prevalence of multi-vessel disease from fairly small cohorts, and thus establishing the prevalence of multi-vessel disease in larger cohorts warrants further investigation.

A possible explanation for the association between the NIHSS score and NSMIA can be proposed. The severe neurological deficit seen in patients with multi-vessel disease may be related to poor collateral circulation and impaired dynamic cerebral autoregulation (DCA), which is known to be severely impaired in patients with bilateral ICA stenosis. Patients with bilateral carotid artery stenosis may have elevated Doppler flow velocities and impaired baroreflex sensitivity [13, 14]. Patients with bilateral vertebral stenosis were also reported to have hypertension and poorer clinical outcomes compared to those with unilateral stenosis [8]. Bilaterally, the ICA and VA are the main source of blood supply to the brain. These four arteries coalesce to form an equalizing distributor, the circle of Willis, which can redistribute blood flow and compensate for any lack in blood flow from the other contributing vessels. The lack of primary collaterals in the circle of Willis leads to a poor hemodynamic status [15]. Therefore, in patients with multiple artery disease, if the capacity of the collaterals is impaired, hemodynamic compensation is insufficient to maintain the function of DCA. Stenosis and occlusion of the ICA can lead to an elevation of blood flow to the VA [16]. In carotid artery disease, it has been shown that the presence of collaterals through the circle of Willis plays an important role in determining stroke severity [17]. The therapeutic potential of these collaterals was thoroughly reviewed in a recent paper [18]. Moreover, our findings of the strong association between NSMIA and SBP also support the proposed theory.

Several characteristics aside from the SBP correlated with the NSMIA in the bivariate correlation analysis. Age is one such factor, and this finding is consistent with a previous report which described the relationship between the number of plaques and advancing age [19]. The underlying mechanism for this phenomenon requires further investigation. Both the univariate analysis and bivariate correlation indicate that the degree of stenosis is not correlated with the NSMIA. Multivariate regression analysis indicated that the degree of stenosis is a significant independent risk factor for stroke severity. The relationship between the degree of stenosis and stroke severity has been discussed in previous studies [20, 21].

In the multivariable linear regression analysis, HDL levels and female gender were significantly and independently associated with ischemic stroke severity. Similar results have been reported in previous studies. A low baseline HDL-C (≤35 mg/dL) at admission was associated with greater stroke severity [22]. This association was also identified in a cohort of younger patients [23]. We also identified female gender as an independent factor associated with stroke severity. This finding is consistent with those of a previous population-based study which showed that the sex difference in stroke severity is independent of age, subtype of stroke, and cardiovascular risk factors [24].

Our research has significant clinical implications. The strong association between the NSMIA and stroke severity indicates that the general symptom of stroke is related to both anterior and posterior circulation stenosis. In contrast with the ICA, VA stenosis has received much less attention [25]. However, up to a quarter of ischemic stroke was related to posterior stenosis in a community-based study [26]. Currently, several noninvasive imaging techniques for the vertebral system are being developed to aid in the assessment of stenosis, including Duplex ultrasound, magnetic resonance angiography and CTA. However, compared to invasive imaging techniques, the sensitivity and specificity of the noninvasive approaches still need further improvement [27]. Given the presence of multilateral stenosis, complete imaging of both the anterior and posterior circulations could be suggested for high risk patients in order to better understand a patient’s unique stenosis profile. Cerebrovascular revascularization has been proven to be beneficial for symptomatic ischemic stroke patients. Patients with multilateral stenosis should be considered as high risk compared to those patients with unilateral or bilateral stenosis. More studies are needed to evaluate the risk-benefit profile of multilateral revascularization and to optimize medical treatment. We suggest that a full stenosis profile, including an assessment of the number of stenotic arteries and the degree of stenosis should be considered as part of the criteria for revascularization therapy in the future.

There are several strengths to our study. Firstly, to the best of our knowledge, this is the first study that illustrates the importance of the number of stenotic arteries in cerebrovascular disease. Secondly, the diagnosis of an ischemic stroke was confirmed in all patients using noninvasive CTA, thus excluding patients with other subtypes of stroke. Thirdly, DSA is still considered as the gold standard for the evaluation of cerebrovascular stenosis and provides the most sensitive and specific assessment of the degree of stenosis. The limitations of this study include its retrospective design and the fact that the stroke severity of all patients was limited to minor and moderate strokes (the median NIHSS was 10, and the maximum NIHSS was 15). Furthermore, since DSA is an invasive imaging technique, this limited the number of patients that we were able to recruit for the study. The number of patients with three or four vessels stenosis was limited and could result in a potential bias. Therefore, more studies with larger cohorts are needed for further validation.

## Conclusions

In conclusion, our study demonstrated that the number of stenotic major intracranial arteries is independently associated with an increase in the severity of ischemic stroke. NSMIA and the degree of stenosis are two factors which are independently associated with the severity of ischemic stroke. We suggest that the number of stenotic major intracranial arteries is a novel factor which should be used in conjunction with the degree to stenosis to assess the severity of ischemic stroke. Taking this factor into account may help us to identify each individual patient’s symptom severity more accurately and to optimize their treatment.
